# OPIOID USE DISORDER AMONG OLDER ADULTS: ADDRESSING MULTIMORBIDITY AND GERIATRIC CONDITIONS

**DOI:** 10.1093/geroni/igac059.650

**Published:** 2022-12-20

**Authors:** Benjamin Han

**Affiliations:** UC San Diego, La Jolla, California, United States

## Abstract

Dr. Han will present multidisciplinary research focused on understanding the healthcare needs of older adults with opioid use disorder. Nationally, there is a sharp increase in older adults with opioid use disorder (OUD) and the number of older adults dying of opioid-related overdoses. This lecture will first describe the prevalence of geriatric conditions and comorbidities among older adults who receive care in opioid treatment programs. Then, Dr. Han will present patterns of acute healthcare utilization among older adults with opioid use disorder. Qualitative data from patient experiences of aging with opioid use disorder and the challenges of managing chronic diseases will then be presented. The lecture will conclude with a discussion about opportunities for developing multidisciplinary models of care to best deliver geriatric-based interventions for this population.

